# Examining the use of a continuous marker of metabolic syndrome severity for detecting resting autonomic dysfunction in a multiracial sample of young adults

**DOI:** 10.3389/fcvm.2025.1508805

**Published:** 2025-03-04

**Authors:** Ta’Quoris A. Newsome, Austin J. Graybeal, Ryan S. Aultman, Anabelle Vallecillo-Bustos, Caleb F. Brandner, Sydney H. Swafford, Abby T. Compton, Sarah Parnell, Rhett C. Schimpf, Tanner Thorsen, Megan E. Renna, Jon Stavres

**Affiliations:** ^1^ School of Medicine, University of Mississippi Medical Center, Jackson, MS, United States; ^2^ School of Kinesiology and Nutrition, The University of Southern Mississippi, Hattiesburg, MS, United States; ^3^ Department of Health and Human Physiology, The University of Iowa, Iowa City, IA, United States; ^4^ School of Psychology, The University of Southern Mississippi, Hattiesburg MS, United States

**Keywords:** metabolic syndrome, baroreflex sensitivity, blood pressure, heart rate variability, risk assessment

## Abstract

**Aims:**

To determine if a continuous marker of metabolic syndrome (MetS) severity (MetS_index_) could identify early-onset autonomic dysfunction in young adults at an elevated risk (ER) of MetS.

**Methods:**

Blood biomarkers and anthropometrics were collected from 178 individuals. Cardiovagal baroreflex sensitivity (cBRS) and heart rate variability (HRV) were evaluated during 10-min of rest. Linear regressions examined the associations between the MetS­_index_ and cBRS, as well as select indices of HRV. These variables were also compared between individuals meeting the criteria for MetS (MetS group), individuals not meeting the criteria for MetS but having a positive MetS_index_ (ER), and healthy controls (Con) matched for sex, race, and ethnicity (*n* = 20 per group).

**Results:**

All indices of cBRS (all *p* ≤ 0.007) and the standard deviation of normal-to-normal r-r intervals (SDNN; *p* = 0.001) were attenuated in the MetS group compared to the Con group. However, no differences were observed between the Con and ER groups (*p* ≥ 0.395). The MetS_index_ did demonstrate a significant, albeit small (R^2^ ≤ 0.038, *β*≤ −0.168, *p* ≤ 0.028) association with all indices of cBRS and SDNN.

**Conclusions:**

The MetS_index_ is associated with indices of cBRS and HRV, but is not currently able to detect early-onset autonomic dysfunction in young adults with an elevated risk of MetS.

## Introduction

Approximately 41.8% of United States (U.S.) adults are affected by metabolic syndrome [MetS (1)], and the prevalence is increasing most rapidly among young adults aged 20–39 (2). This is especially alarming when considering that the risk of developing MetS increases by approximately 50% for every decade increase in age (3). Thus, as medical advancements continue to extend the average life expectancy in the U.S., the prevalence and severity of MetS related chronic diseases are expected to increase considerably. As such, it is important for both researchers and clinicians to gain a comprehensive understanding of the pathophysiology and progression of this condition to develop effective mitigation strategies in young adults.

Recent literature has placed a growing emphasis on understanding the progression of autonomic dysfunction in individuals with MetS (4). For instance, studies have reported attenuated heart rate variability (HRV) (5, 6), elevated sympathetic tone (7, 8), and exaggerated exercise pressor responses (9) in individuals with MetS. Prior studies have also demonstrated MetS related impairments in baroreflex control (10, 11), a factor that likely contributes to exaggerated blood pressure values both at rest and during exercise. This raises questions regarding the sequence of progression for both autonomic dysfunction and MetS. For example, it is unclear whether impairments in autonomic function develop in a progressive manner concurrent to the accumulation of MetS components. Currently, MetS diagnoses are dichotomous, categorizing individuals as either having or not having MetS. However, if autonomic dysfunction does occur in a progressive manner alongside worsening MetS components (i.e., hypertension, hyperglycemia, obesity, and dyslipidemia), this binary classification may overlook a subset of individuals who are still at an elevated risk of cardiometabolic dysfunction despite not meeting the formal criteria for a MetS diagnosis. Subsequently, researchers have developed a continuous marker that quantifies an individual's average risk for developing cardiometabolic disease relative to the average U.S. citizen, known as the continuous MetS severity score (MetS_index_) (12). If autonomic function does progressively develop with MetS, this score can be a valuable tool for early detection. Accordingly, this study tested the hypothesis that young adults between the ages of 18 and 39 with an elevated MetS_index­_ would demonstrate significantly lower spontaneous cardiovagal baroreflex gain (cBRS; a closed-loop index of resting baroreflex control) and heart rate variability (HRV) compared to control participants. If true, this would support the use of a continuous index of cardiometabolic disease risk for detecting early-onset autonomic dysfunction in young adults. Considering that the MetS_index­_ equations were originally developed using data from a predominantly older demographic (12), we also tested a secondary equation based on sample-specific z-scores (13), allowing for a more age-specific assessment of MetS risk.

## Materials and methods

### Subjects and study design

A total of 178 individuals between the ages 18 and 39 years old (Age: 22 ± 4 years; BMI: 26.4 ± 6.6 kg/m^2^) originally completed this two-visit study protocol at the University of Southern Mississippi. To be included in this study, participants must have been between eighteen and thirty-nine years of age, not pregnant or lactating, and be free of any known cardiovascular, metabolic, pulmonary, renal, or neurological diseases. These included diagnosed type 2 diabetes, and excluded the hypertensive component of MetS. Among these, thirty-seven (20.8%) met the National Cholesterol Education Program (NCEP) Adult Treatment Panell III (ATP III) guidelines for MetS, which included any combination of three of the following five risk factors: (1) a waist circumference (WC) ≥ 102 cm for males and ≥88 cm for females (≥80 cm for Asian females and ≥90 cm for Asian males), (2) a fasting blood glucose (FBG) reading ≥100 mg/dl, (3) a fasting triglyceride (TRG) reading ≥150 mg/dl, (4) a systolic blood pressure (SBP) reading ≥130 mmHg or a diastolic (DBP) reading ≥85 mmHg, or (5) a high-density lipoprotein cholesterol (HDL-C) reading <40 mg/dl for males or <50 mg/dl for females. HbA1C was also collected from a subset (*n* = 58) of individuals in addition to FBG, and any individual taking medications to treat any of the risk factors mentioned above was counted as having that risk factor. The participants who presented with <3 risk factors but a positive metabolic syndrome severity score (described in more detail below) were placed in the elevated risk (ER) group. All protocols were approved by the University of Southern Mississippi Institutional Review Board (IRB# 22-1012 and 23-0446), and all participants were provided written informed consent.

### Anthropometrics and body composition assessments

In visit 1, a standard stadiometer and calibrated digital scale were used to measure height and weight, respectively, for each participant. WC was collected at the iliac crest using a spring-loaded, flexible aluminum tape measure (14). Total body fat (BF%), fat mass (FM), and fat-free mass (FFM) were also assessed via bioelectrical impedance spectroscopy (BIS; SFB7, ImpediMed, Carlsbad, CA, USA) as described previously (15–17), and resting blood pressure measurements were recorded using an automated sphygmomanometer (OMRON healthcare inc., Sunrise, FL) following a five-minute rest period.

### Fasting blood glucose and lipids

Following a minimum eight hour fast and a twenty-four hour abstention from exercise, a 40-microliter blood sample was collected via a traditional capillary fingerstick and analyzed using a point-of-care lipid analyzer [Cholestech LDX, Abott, Abott Park, IL (18)]. This device yielded readings of HDL-C, low-density lipoprotein cholesterol (LDL-C), total cholesterol (TC), TRG, and FBG. Notably, this device does not report HDL-C readings < 5 mg/dl or TRG values >650 mg/dl or <45 mg/dl. Instead, these values are expressed as 15 mg/dl, 650 mg/dl, or 45 mg/dl, respectively, leading to over- or underestimation in such cases. Consequently, LDL-C was unable to be calculated for participants who demonstrated values outside of the detectable ranges (sample sizes for LDL-C, HDL-C, TRG, and TC provided as footnotes in Table 1). HbA1C was collected using an automated HbA1C analyzer [A1CNow^+^, Pts Diagnostics, Whitestown, IN (19)].

**Table 1:** Participant demographics and metabolic syndrome criteria for the study sample, and compared across MetS risk groups.

Variable description	Entire sample	Matched groups			
		Con	ER	MetS	*p*
Demographics	*n* = 171	*n* = 20	*n* = 20	*n* = 20	
M/F (n)	70/101	12/8	12/8	12/8	
White/BAA/A NA (n)	72/60/38/1	6/9/5/0	6/9/5/0	6/9/5/0	
Age (yrs)	22 ± 4	22 ± 4	23 ± 5	23 ± 5	0.774
Height (cm)	168.2 ± 9.0	168.6 ± 10.2	170.0 ± 9.5	174.3 ± 10.0	0.180
Weight (kg)	75.4 ± 22.2	71.2 ± 16.6	86.6 ± 19.2	101.4 ± 33.6^e^	0.001
BMI (kg/m^2^)	26.4 ± 6.6	24.8 ± 4.1	30.1 ± 7.4^e^	33.2 ± 10.1^e^	0.004
LDL-C (mg/dl)^a^	90 ± 23	90 ± 24	83 ± 26	102 ± 24	0.351
TC (mg/dl)^b^	154 ± 34	150 ± 28	141 ± 28	182.2 ± 48.0†	0.032
MetS Criteria					
WC (cm)	87.4 ± 15.4	83.7 ± 12.3	95.9 ± 16.8	105.2 ± 21.3^e^	<0.001
FBG (mg/dl)	89 ± 8	88 ± 6	89 ± 6	97 ± 10^e^^,^^f^	0.001
SBP (mmHg)	115 ± 13	116 ± 12	116 ± 12	127 ± 13^e^^,^^f^	0.009
DBP (mmHg)	79 ± 10	78 ± 8	75 ± 7	90 ± 11^e^^,^^f^	<0.001
TRG (mg/dl)	118 ± 109	75 ± 32	180 ± 149^e^	204 ± 175^e^	0.008
HDL-C (mg/dl)^c^	47 ± 14	51 ± 13	38 ± 13^e^	33 ± 9^e^	<0.001
HbA1C (%)^d^	4.96 ± 0.44	4.95 ± 0.38	4.85 ± 0.37	5.33 ± 0.63	0.072
MetS Severity					
MetS_index_ (a.u.)	−0.30 ± 0.77	−0.75 ± 0.42	0.38 ± 0.30^e^	0.83 ± 0.63^e^^,^^f^	<0.001
MetSz (a.u.)	−0.00 ± 0.47	−1.64 ± −0.31	0.18 ± 0.29^e^	0.63 ± 0.44^e^^,^^f^	<0.001
cBRS and HRV					
cBRS_all_	22.6 ± 12.9	25.7 ± 9.7	23.1 ± 10.1	14.8 ± 8.8	0.002
cBRS_up_	28.6 ± 18.9	34.4 ± 20.7	28.9 ± 15.5	17.7 ± 12.5	0.008
cBRS_down_	17.9 ± 9.6	20.2 ± 8.2	18.2 ± 7.4	12.6 ± 7.0	0.007
SDNN	89.8 ± 39.6	106.3 ± 42.6	93.7 ± 35.7	62.2 ± 31.7	0.001
RMSSD	72.3 ± 42.8	87.0 ± 52.1	75.1 ± 32.7	46.3 ± 32.4	0.007
LF/HF Ratio	7.1 ± 5.7	8.6 ± 9.0	5.8 ± 4.2	6.9 ± 5.7	0.424

CON, healthy controls; ER, elevated metabolic syndrome risk; MetS, metabolic syndrome group; M, male; F, female; BMI, body mass index; LDL-C, low-density lipoprotein cholesterol; TC, total cholesterol; WC, waist circumference; FBG, fasting blood glucose; SBP, systolic blood pressure; DBP, diastolic blood pressure; TRG, triglycerides; HDL-C, high-density lipoprotein cholesterol; HbA1C, glycated hemoglobin; MetS­_index_, metabolic syndrome severity score originally developed by Gurka and colleagues (2014); MetS*z*, modified metabolic syndrome severity score based on younger age group; cBRS_all,_ spontaneous cardiovagal baroreflex gain of all baroreflex sequences; cBRS_up,_ cBRS of all up-ramping sequences; cBRS_down,_ cBRS of all down-ramping sequences; SDNN, standard deviation of normal-to-normal r-r intervals, RMSSD, root-mean square of successive differences in normal-to-normal r-r intervals; LF/HF ratio, low-frequency to high-frequency ratio

^a^ (*n* = 44).

^b^ (*n* = 57).

^c^ (*n* = 170).

^d^ (*n* = 56).

^e^ significantly different from CON.

^f^ significantly different from ER.

### Metabolic syndrome risk scores (MetS_index_ and MetS_Z_)

Along with the dichotomous classification of MetS, the entire sample of participants were organized based on two separate continuous MetS severity scores. One of these scores (MetS_index_), which was originally published by Gurka et al. (12), expands on the binary classification of MetS by weighting the severity of each individual component of MetS (i.e., the actual values for resting SBP, FBG, TRG, HDL, and WC), and is derived from equations specific to a person's sex, race, and ethnicity. Due to the lack of published equations for Asian males and females (12), the MetS_index_ was calculated using the sex-specific non-Hispanic White equations for all Asian participants. The resultant score ranges from −5.0 a.u. (demonstrating minimal relative MetS severity) to +5.0 a.u. (representing the highest possible severity of MetS), and a score of 0.0 a.u. represents the average MetS severity of the sample population [*n* = 6,870, age range 20–64 years (12);]. The benefit of this scoring system is that it may effectively detect individuals who are at an elevated risk of MetS, as demonstrated by a positive score, even without meeting the ATP III criteria for MetS. Based on this approach, the participants were separated into three groups: individuals who did not meet the formal ATP III criteria for MetS and had a negative MetS_index_ score (Con), those who did not meet the formal ATP III criteria for MetS but had a positive MetS_index_ score (Elevated Risk; ER), and those who met the formal ATP III criteria for MetS and had a positive MetS_index_ score (MetS). Individuals who met the formal criteria but had a negative MetS_index_ score were classified as “MetS+Low Risk” and were excluded from the matched-groups and regression analyses (see below for more details).

In addition to the MetS_index_, this study also investigated the use of a new secondary score that was developed using a more narrowly focused study sample of young adults [MetS*z* (13)]. While the MetS_index_ holds important clinical potential for the general population, the inclusion of older adults (up to 64 years of age) in the development of the MetS_index_ may result in an underestimation of age-specific cardiometabolic risk in younger adults (20–39 years), thereby masking underlying risk. The newly developed MetS*z* score may circumvent this problem by averaging the z-scores for each individual risk factor for males and females separately, as described in the following equation.

MetSz=(WCz+SBPz+[ln]TRGz+FBGz–[ln]HDL-Cz)/5

In this equation, WC*z* represents the sex-specific z-score for waist circumference, SBP*z* represents the z-score for SBP for the entire sample, [ln]TRG*z* represents the z-score for the log transformed TRG for the entire sample, FBG*z* represents the z-score for FBG for the entire sample, and [ln]HDL-C*z* represents the sex-specific z-score for log transformed HDL-C values. Similar to the MetS­_index_, any participant who did not meet the NCEP ATP III criteria for MetS and had a MetS*z* score below the 50th percentile was classified as healthy (Con), whereas participants who did not meet the NCEP ATP III criteria but had a MetS*z* score at or above the 50th percentile was assigned to the ER group, and individuals meeting the NCEP ATP III criteria who had a MetS*z* score at or above the 50th percentile was assigned to the MetS group. Likewise, individuals meeting the NCEP ATP III criteria who had a MetS*z* score below the 50th percentile were classified as MetS+Low Risk and were excluded from the matched pairs and regression analyses. It is important to note that the z-scores used to calculate the MetS*z* were calculated using the entire sample (*n* = 178), and these values were not adjusted after excluding individuals classified as MetS+Low Risk.

### Cardiovagal baroreflex sensitivity and heart rate variability assessments

Cardiovagal baroreflex sensitivity (cBRS) was assessed during ten minutes of supine rest during the second visit. Throughout this period, heart rate and beat-by-beat blood pressure was recorded using a one-lead electrocardiogram (Lead I; PowerLab, AD Instruments, Colorado Springs, CO) and finger photoplethysmography (Finapres Nano, AD Instruments, Colorado Springs, CO), respectively. Breathing frequency was regulated by a metronome set to a cadence of seven breaths per minute. This breathing frequency was chosen based on prior evidence that cardiac vagal tone is maximized at approximately six breaths per minute (20), and likewise, pilot testing determined that a cadence of seven breaths per minute was the slowest comfortable breathing rate. Following exclusion of ectopic beats, SBP and cardiac interval were extracted on a beat-by-beat basis and used to calculate cBRS via the sequence method. Based on previous recommendations (21), the minimum length for all cBRS sequences was set to 3, with a delay of 1 beat, minimum thresholds of 1 mmHg and 5 ms for SBP and cardiac interval changes, respectively, and a minimum correlation coefficient *r* ≥ 0.8 [CardioSeries v2.7 (22)]. In addition to reporting total baroreflex gain for all combined sequences (cBRS_all_), the baroreflex gain of all up-ramping SBP sequences (cBRS_up_) and all down-ramping SBP sequences (cBRS_down_) were reported independently. Select indices of HRV were also reported from this same period of data. These measures included the standard-deviation of normal-to-normal cardiac intervals (SDNN), the root-mean-square of successive normal-to-normal intervals (RMSSD), and the ratio of normalized low-frequency to normalized high-frequency components of HRV (LF/HF ratio). Historically, lower SDNN and RMSSD, and higher LF/HF ratios, have been associated with higher sympathetic to parasympathetic tone. However, more recent evidence challenges this notion and suggests that both sympathetic and parasympathetic activity contributes to changes in these indices, with RMSSD being influenced by vagal tone to a greater degree (23).

### Statistical approach

Data were first inspected for potential outliers using histograms and boxplots. Upon confirmation of no outliers, three separate sub-analyses were conducted. First, the utility of the MetS_index_ was examined using a combination of linear regression analyses, which predicted individual markers of cBRS and HRV using the MetS­_index­_. Importantly, these analyses were conducted using the entire study sample (*n* = 171), with only the individuals classified as MetS+Low Risk excluded (*n* = 7; 3.9%). In addition to linear regression analyses, measures of cBRS and HRV were also compared across the MetS_index_ assigned Con, ER, and MetS groups using a matched-groups analysis. For this matched-groups analysis, individuals in the MetS group were matched to individuals in the ER and Con groups by sex, race, and ethnicity, and cBRS and HRV values were compared across groups using a one-way analysis of variance (ANOVA) employing a Tukey *post-hoc* correction for multiple comparisons. Frequency analyses were then used to examine the number of participants who were reclassified across groups when using the MetS*z* scoring system compared to the MetS_index_­, and general linear models examined the association between the two scores (MetS­_index_ vs. MetS*z*) while including the MetS_index_ assigned risk group as a factor (this time including the MetS+Low Risk group). Using MetS­_index_ as the reference model, Bland-Altman analyses were also used to determine the constant bias (i.e., mean difference) and 95% limits of agreement between MetS indices, and linear regression techniques were used to determine proportional biases. Following these analyses, linear regressions were repeated to examine the association between the MetS*z* score and indices of cBRS and HRV. All statistical analyses were conducted using Jamovi software (version 2.3.21.0), and significance was accepted at *p* < 0.05.

## Results

### Level of agreement between MetS_index_ and MetSz scores

**Figure 1:**
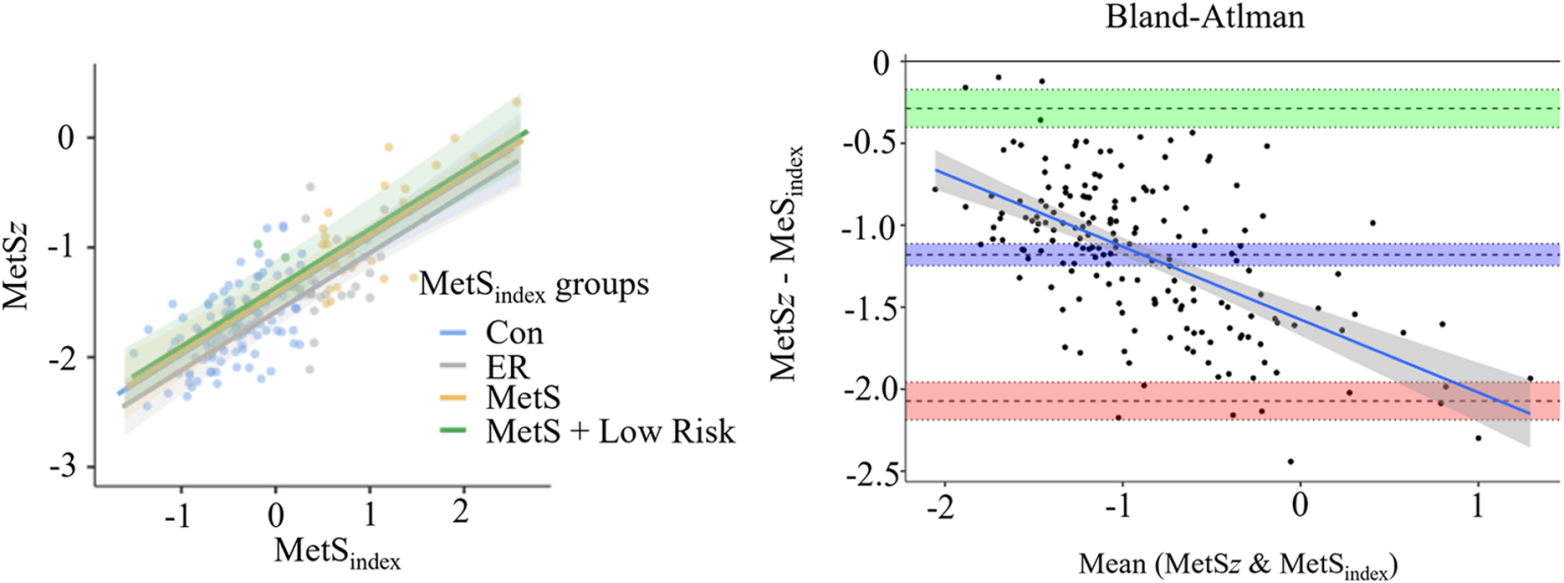
Agreement between the MetS_index_ and MetS*z* scoring systems. The left panel displays the association between the MetS_index_ and MetS*z* scores across each MetS_index_ based risk group using general linear modeling, and the right panel displays a Bland-Altman plot comparing the level of agreement between the two scoring systems.

Participant demographics are presented in **Table 1**. Of the 171 participants included in the single group analysis, 59.1% were female, 40.9% were male, 5.3% were Hispanic, 94.7% were non-Hispanic, 42.1% were White, 35.1% were Black or African American, 22.2% were Asian, and <1% were Native American. As shown in Figure 1, the MetS*z* scores demonstrated a strong association (*R*^2^ = 0.699, *p* < 0.001) with the original MetS_index_. Likewise, a Bland-Altman analysis indicated a mean difference of 0.300 a.u. (95% CI: 0.232/0.367) with limits of agreement ranging from −0.595 (95% CI: −0.710 −0.479) to 1.194 (1.079 1.310) and a significant proportional bias (*R*^2^ = 0.453, *p* < 0.001). Consistent with these levels of agreement, every participant was classified into the same MetS risk group using both scores, meaning that no participant was considered to have a higher or lower risk when using the MetS­*z* score compared to the MetS_index_. Because of this, a matched-groups analysis was not performed for the MetS*z* score.

### Evaluating the use of the MetS_index_

When evaluating MetS groups based on the MetS_index­_, 67.8% were classified as healthy (Con), while 14.6% were classified as ER and 17.5% were classified into the MetS group. When conducting the matched-groups analyses using the MetS_index_ score, a total of sixty individuals could be matched across all three groups by sex, race, and ethnicity (twenty per group). As expected, individuals in the MetS group were significantly heavier, had a higher BMI, WC, FBG, SBP, DBP, TRG, MetS_index_, MetS*z*, and lower HDL-C values compared to the Con group (all *p* ≤ 0.022). The MetS group also had a higher TC, FBG, SBP, DBP, MetS­_index­_, and MetS*z* compared to the ER group (all *p* ≤ 0.045). Likewise, the ER group had a higher BMI, TRG, MetS_index_, MetS*z*, and a lower HDL-C value compared to the Con group (all *p* ≤ 0.043). No significant differences were observed for age, height, LDL-C, or HbA1C (*p* ≥ 0.072).

**Figure 2:**
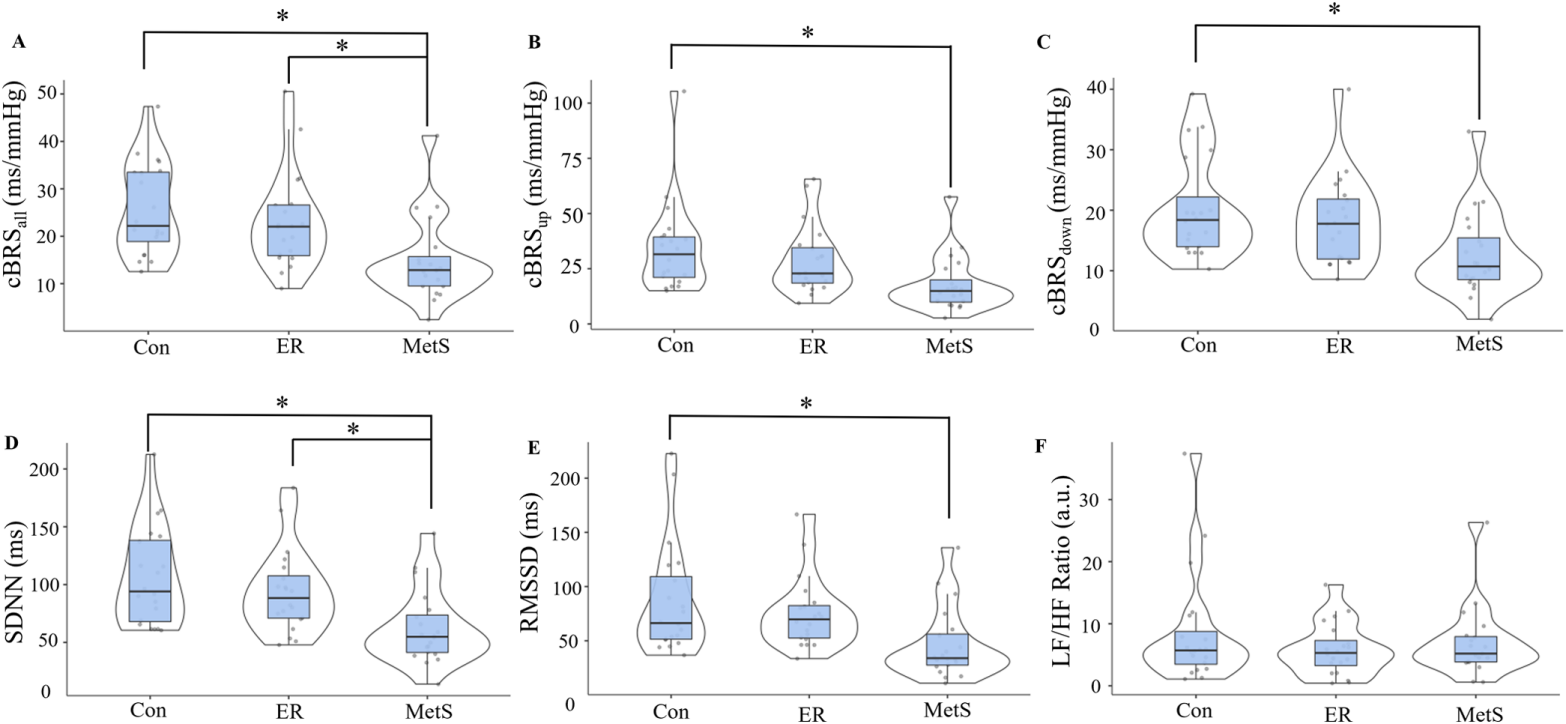
Cardiovagal baroreflex sensitivity (cBRS) for all sequences [cBRS_all_; **(A)**], all up-ramping sequences [cBRS_up_; **(B)**], and all down-ramping sequences [cBRS_down_; **(C)**], as well as the standard-deviation of all normal-to-normal cardiac intervals [SDNN; **(D)**], the root-mean-square of all successive normal-to-normal cardiac cycles [RMSSD; **(E)**], and the ratio of normalized low-frequency to high-frequency heart rate variability components [LF/HF ratio; **(F)**] compared across control (Con), elevated risk (ER), and metabolic syndrome (MetS) groups according to the continuous marker of metabolic syndrome severity (MetS_index_) originally published by Gurka et al. (12). *indicates a statistically significant difference (*p* < 0.05).

Results from this matched pairs analysis are presented in Figure 2. Significant main effects of group were observed for cBRS_all_ (*F* = 7.143, *p* = 0.002), cBRS_up_ (*F* = 5.238, *p* = 0.008), cBRS_down_ (*F* = 5.426, *p* = 0.007), SDNN (*F* = 7.553, *p* = 0.001), and RMSSD (*F* = 5.438, *p* = 0.007). These main effects were explained by significantly attenuated cBRS_all_ in the MetS group compared to both the Con (mean diff = −10.93, *p* = 0.002) and ER groups (mean diff = −8.31, *p* = 0.021), significantly attenuated cBRS_up_ in the MetS group compared to the Con group (mean diff = −16.7, *p* = 0.007), significantly attenuated cBRS_down_ in the MetS group compared to the Con group (mean diff = −7.61, *p* = 0.007), significantly attenuated SDNN in the MetS group compared to both the Con (mean diff = −44.1, *p* = 0.001) and ER groups (mean diff = −31.5, *p* = 0.025), and significantly attenuated RMSSD in the MetS group compared to the Con group (mean diff = −40.7, *p* = 0.006). However, no measures of cBRS or HRV were significantly different between the Con and ER groups (all *p* ≥ 0.395). When the entire sample (*n* = 171) was examined using linear regression analyses, results indicated small, but statistically significant associations between the MetS_index_ and cBRS_all_, cBRS_up_, cBRS_down_, and SDNN (Table 2)._­­_ Like the MetS_index_, regression analyses revealed weak, but statistically significant associations between the MetS*z* score and cBRS_all_, cBRS_up_, cBRS_down_, and SDNN (*p* ≤ 0.039), but not RMSSD or the LF/HF ratio (*p* ≥ 0.138; Table 2).

**Table 2:** Linear regression analyses results.

Variable description	cBRS_all_			cBRS_up_			cBRS_down_		
	R^2^	*p*	*β*	R^2^	*p*	β	R^2^	*p*	β
MetS_index_	0.038*	0.011	−0.195	0.032*	0.018	−0.182	0.036*	0.012	−0.192
MetS*z*	0.036*	0.013	−0.190	0.030*	0.024	−0.172	0.033*	0.018	−0.181
	SDNN			RMSSD			LF/HF		
	R^2^	*p*	β	R^2^	*p*	β	R^2^	*p*	β
MetS_index_	0.028*	0.028	−0.168	0.017	0.088	−0.131	0.002	0.543	−0.047
MetS*z*	0.024*	0.039	−0.158	0.013	0.138	−0.114	<0.001	0.683	0.032

* statistically significant association (*p* < 0.05); MetS­_index_, metabolic syndrome severity score originally developed by Gurka and colleagues (2014); MetS*z*, modified metabolic syndrome severity score based on younger age group; cBRS_all_, cardiovagal baroreflex sensitivity of all baroreflex sequences; cBRS­_up_, cardiovagal baroreflex sensitivity of all up-ramping blood pressure sequences; cBRS_down­_, cardiovagal baroreflex sensitivity of all down-ramping blood pressure sequences; SDNN, standard-deviation of normal-to-normal cardiac cycles; RMSSD, root mean square of successive differences in normal-to-normal cardiac cycles; LF/HF, ratio of low-frequency to high-frequency components of heart rate variability. *n* = 171.

## Discussion

This study tested the hypothesis that young adults at an elevated risk of MetS, as indicated by a continuous marker of MetS severity [the MetS_index_ (12);] would demonstrate attenuated cBRS and HRV values relative to healthy control participants. The results of this study revealed a significant effect of group for cBRS_all_, cBRS_up_, cBRS_down_, SDNN, and RMSSD using the MetS_index_, however, this was primarily driven by decreases in cBRS and HRV indices in the MetS group. In contrast, individuals in the ER group did not demonstrate significantly lower cBRS or HRV values relative to the control group. There were also weak but significant associations between the MetS_index_ and all indices of cBRS and SDNN, which were also observed when using the modified MetS*z* score. Taken together, these findings suggest that the MetS_index_ shows viability as a valid predictor of metabolic dysfunction, but may not be sensitive to early autonomic impairments in young adults. Some possible explanations for this are discussed in the following sections.

### Detecting autonomic dysfunction using a continuous marker of cardiometabolic disease risk

The observation that cBRS and HRV values were attenuated in the MetS group compared to the Con group agrees with previous studies showing significant impairments in both closed loop (11) and open loop (10) assessments of baroreflex function in individuals with MetS. In the study by Dutra-Marques et al. (11), investigators compared spontaneous cBRS and MSNA at rest between individuals with a normotensive phenotype of MetS (*n* = 27) and control participants without MetS (*n* = 27), and found that spontaneous cBRS was reduced and resting MSNA was elevated in the MetS group. Likewise, Grassi et al. (10) compared sympathetic BRS and resting MSNA between individuals with (*n* = 48) and without (*n* = 43) MetS, and found significant increases in resting MSNA and reductions in sympathetic BRS in the MetS group. Therefore, despite the limited sample sizes in the between-group comparison (*n* = 20 per group), the results of the present study generally agree with similar work by demonstrating MetS related impairments in cBRS and HRV.

Several mechanisms have been proposed to mediate this impairment, the primary of which include a combination of hyperglycemia (24), insulin resistance (25, 26), and remodeling of the carotid artery (26). Importantly, these adaptations would not be assumed to simply appear with the formal diagnosis of MetS, but rather, would likely develop in a progressive manner in conjunction with increasing cardiometabolic disease risk. It was this very consideration that led to the hypothesis that a continuous marker of MetS severity (the MetS_index_) would be able to detect early onset baroreflex dysfunction and impaired cardiac autonomic tone in young adults. However, contrary to this hypothesis, young adults classified as elevated risk in this study did not demonstrate a significant decrease in cBRS or any differences in markers of HRV compared to healthy control participants, and the MetS­_index_ only demonstrated a weak relationship with markers of autonomic function. We consider two likely explanations for these findings. The first explanation is that the progression of cardiometabolic dysfunction accelerates as an individual approaches a formal MetS diagnosis, which is reflected by a similar acceleration in autonomic dysfunction. This would result in an apparent “onset” of baroreflex dysfunction coinciding with a formal diagnosis. This notion is supported by the interactions between each of the defined MetS risk factors. For example, hyperglycemia, a primary component of MetS, directly contributes to insulin resistance over time (27, 28), and in turn, insulin resistance exaggerates hyperglycemia. Both of these factors are suggested to independently influence sympathetic activity and blood pressure (29–31), and increases in resting sympathetic activity can lead to increases in circulating catecholamines and glucagon (32), further exacerbating hyperglycemia. These interactions would presumably lead to an exponential increase in cardiometabolic disease risk, rather than a linear increase, and may therefore explain why impairments in cBRS and HRV were observed in the MetS group, but not the ER group. However, while the differences between the Con and ER groups were not statistically significantly different, the group mean responses for cBRS­_all_, cBRS_up_, and SDNN did trend towards a net decrease in the ER group compared to the Con group. As such, it may also be possible that the subtle differences do exist in these values in individuals who are at an elevated risk of MetS, but the MetS_index_ is simply not sensitive enough to detect these changes. This notion is supported by the observed weak, but significant associations between both MetS risk scores (the MetS­_index_ and the MetS*z* score) and cBRS and SDNN (Table 2). If true, other methods of assessing autonomic function, such as open loop assessments of cardiac and sympathetic baroreflex control, may provide a more sensitive evaluation of early-onset autonomic dysfunction in young adults.

### Practical implications

As noted previously, the findings of this study did not support the hypothesis that individuals classified as having an elevated risk of MetS according to the MetS_index_ would demonstrate impaired cBRS and HRV. As such, we cannot conclude that the MetS_index_ is a valuable method of detecting early onset-autonomic dysfunction in young adults. While these findings may limit the utility of the MetS_index_ for this purpose, we caution readers that the overall value of this continuous marker of MetS severity should not be discarded altogether. Despite the lack of group differences, the MetS_index_ still demonstrated a statistically significant, albeit weak, relationship with indices of cBRS and HRV. Therefore, while the clinical relevance of this score may be currently limited, the observation of a statistically significant relationship indicates that further improvements to this score may be possible. This also supported by prior studies demonstrating associations between the MetS_index_ and eating behaviors (33) and visceral adiposity (34). With this in mind, future studies may consider evaluating additional MetS related determinants of autonomic function, which may be incorporated into the calculation of the MetS_index_ in an effort to improve both sensitivity and specificity. Furthermore, we also cannot discount the possibility that more sensitive measures of autonomic function, such as pharmacological tests of baroreflex control that incorporate MSNA, would demonstrate stronger associations with the MetS_index_.

### Limitations

This project was subject to certain limitations that warrant further discussion. First, this study only evaluated resting closed-loop (spontaneous) assessments of baroreflex function and HRV and did not collect muscle sympathetic nerve activity (MSNA). This is important, as resting assessments of HRV and BRS are not able to inform dynamic responses to known stressors (such as orthostatic challenges or exercise). Future studies may consider utilizing MSNA recordings, combined with pharmacological stressors (i.e., the modified Oxford method) to evaluate sympathetic BRS responses, providing a more complete picture of resting autonomic function. Second, this study was completed as part of two larger ongoing projects, and therefore, the study sample was determined via convenience sampling. However, power analyses indicate that, using an assumed large effect size of *f* = 0.40 (based on the anticipated differences in cBRS between the MetS and Con groups), sixty-three participants (twenty-one per group) would be required to observe statistically significant main effects using a one-way ANOVA with three separate groups and a desired power of 0.8 [conducted using G*Power version 3.1.9.7 (35)]. Therefore, we remain confident that our study was appropriately powered for these analyses, particularly for the comparisons of cBRS and HRV measures across MetS risk groups.

## Conclusions

This study tested the hypothesis that resting cBRS and HRV indices would be impaired in individuals with an elevated risk of MetS compared to healthy control participants matched for sex, race, and ethnicity. Our findings did not support this hypothesis. Specifically, while individuals with MetS demonstrated the expected decreases in cBRS and HRV compared to healthy controls when grouped by the MetS_index_, these attenuations were not observed in individuals classified as having an elevated risk of MetS. These findings indicate that the progression of resting autonomic dysfunction, as assessed by cBRS and HRV, does not progress linearly with MetS risk, but rather presents alongside the dichotomous classification of MetS. However, considering that both the MetS_index_ and the MetS*z* scores demonstrated a small but statistically significant relationship with cBRS and the SDNN index of HRV, future studies may consider evaluating additional MetS related determinants of autonomic dysfunction for inclusion in the calculation of the MetS_index_. This approach may result in a significantly improved score that is more effective at detecting early onset autonomic dysfunction in individuals at an elevated risk of cardiometabolic disease.

## Data Availability

The raw data supporting the conclusions of this article will be made available by the authors, without undue reservation.
